# Mixing Enhancement in Serpentine Micromixers with a Non-Rectangular Cross-Section

**DOI:** 10.3390/mi9030107

**Published:** 2018-03-02

**Authors:** Joshua Clark, Miron Kaufman, Petru S. Fodor

**Affiliations:** Department of Physics, Cleveland state University, 2121 Euclid Avenue, Cleveland, OH 44236, USA; j.a.clark17@vikes.csuohio.edu (J.C.); m.kaufman@csuohio.edu (M.K.)

**Keywords:** passive micromixers, Dean flows, serpentine-shaped channels, mixing index

## Abstract

In this numerical study, a new type of serpentine micromixer involving mixing units with a non-rectangular cross-section is investigated. Similar to other serpentine/spiral shaped micromixers, the design exploits the formation of transversal vortices (Dean flows) in pressure-driven systems, associated with the centrifugal forces experienced by the fluid as it is confined to move along curved geometries. In contrast with other previous designs, though, the use of non-rectangular cross-sections that change orientation between mixing units is exploited to control the center of rotation of the transversal flows formed. The associated extensional flows that thus develop between the mixing segments complement the existent rotational flows, leading to a more complex fluid motion. The fluid flow characteristics and associated mixing are determined numerically from computational solutions to Navier–Stokes equations and the concentration-diffusion equation. It is found that the performance of the investigated mixers exceeds that of simple serpentine channels with a more consistent behavior at low and high Reynolds numbers. An analysis of the mixing quality using an entropic mixing index indicates that maximum mixing can be achieved at Reynolds numbers as small as 20 in less than four serpentine mixing units.

## 1. Introduction

The use of microfluidic devices in applications ranging from chemical analysis and reaction engineering to biological assays and bioengineering has progressed dramatically in recent years [1,2,3]. This progress has been fueled by the perceived benefits of employing microfluidic devices; such benefits include reduced reactant consumption, superior heat and mass transfer efficiency enabling increased flexibility in reactor or assay design, field deployability, and scalability [1,4,5,6]. Their potential for parallel processing has led to new applications in molecular diagnosis [7] and single cell biology [8,9,10,11,12]. Together with the availability of new methodologies for device fabrication, ranging from soft-lithography [13] to 3D printing [14], laser-assisted chemical etching [15], and even paper-based materials [16], this has encouraged an increasing number of researchers to explore this platform and seek new applications.

One of the fundamental operations that microfluidic devices have to achieve as part of their functionality is mixing. Virtually all their applications, including biological/chemical assays, as well as chemical and particulate analysis, require the mixing of two or more component pairs such as analyte/assay or chemical reactants [17]. Since microfluidic devices operate in the low Reynolds number regime, the typical flow characteristics are laminar, with turbulence being absent; thus, the mixing has to rely on diffusional transport. However, this is too slow for many of the envisioned practical applications. The challenge of achieving efficient mixing in microfluidic devices has spurred a large body of research focused on the theoretical, implementation, and fabrication aspects associated with mixing on the microscale. Nguyen [18], Nguyen and Wu [19], Cai et al. [20], and Lee et al. [17] have provided comprehensive reviews of the various strategies employed to address the mixing bottleneck in the development of microfluidic platforms. In brief, micromixers are generally classified as active or passive. The active micromixers use external energy sources such as ultrasonic [21] or acoustic [22,23,24,25,26] vibration, electric fields [27], magnetic stirrers [28], or mechanical actuators [29] in order to stir the fluids of interest. Configurations such as acoustic-based micromixers have been successfully used to achieve rapid mixing, even when highly viscous solutions are involved [25]. Passive micromixers, on the other hand, use only the interaction between the fluid flow and geometrical structures to sequentially laminate and braid the fluids to be mixed or generate cross-sectional mass transport. The first approach relies on increasing the area of contact between the different fluid components and thus on increasing the efficiency of the molecular diffusion mechanism for mixing [30]. The second approach uses an array of geometrical features, such as ridge/groove systems [31,32,33], obstacles [34], barriers [35], and 2D [36] or 3D [37] turns, to induce transversal advection. It has been shown that, in this case, if the advection induced is chaotic, very fast intermixing between different components can be achieved [18].

While active micromixers can achieve high mixing efficiencies and mixing control over a broad range of Reynolds numbers, they are harder to fabricate, are more difficult to integrate with other microfluidic components, and more importantly require external power sources [20]. Even though in some cases employing complex 3D geometrical structures for mixing control can pose fabrication challenges, for the most part passive micromixers do not suffer from the drawbacks of the active micromixers mentioned above. The absence of an external energy source, aside from the pressure-driven flow, makes them easier to integrate within complex microfluidic systems with standard fabrication methodologies. Moreover, the absence of complex multi-physics interactions that need to be accounted for makes them much more amenable to theoretical or computational modeling. This allows for a more straightforward and efficient optimization process of the various geometrical and flow parameters needed to maximize mixing within various designs [32,38].

A popular design strategy used in passive micromixers to generate cross-sectional flows and induce chaotic advection capable of enhancing the mixing of fluid components, has been the use of channels with repeating curved sections or turns [18]. These systems exploit the centrifugal forces experienced by the fluid as it is guided by the geometry of the channels to move along a curved trajectory. An analysis, first performed by Dean [39], has shown that the flow field that develops inside such a system is consistent with the formation of transversal vortices, also known as Dean flows. These provide a geometrically simple way of promoting advective transport in microchannels using serpentine or spiral-shaped designs. Beside the easy implementation of these designs, additional advantages include the absence of high local shears compared to obstacle-based micromixers as well as the lack of complex 3D surface structures. The first makes them attractive for biological applications targeted at handling without damage to large biomolecules [40]. The second one allows the potential reuse of the devices, as it enables easier cleaning [41]. It has to be noted though that rapid mixing in simple serpentine/spiral designs is achieved typically at large fluid speeds associated with the formation of secondary Dean vortices, leading to the transition to a chaotic advection regime [18]. This corresponds though to Reynolds numbers typically too high for practical settings [42]. In order to achieve more efficient mixing at intermediate and low Reynolds numbers in serpentine micromixers, the effect of various geometrical modifications has been explored. These include the use of grooves on the side or bottom walls of the channel to produce more complex transversal flows [43,44], modulation of the surface of the side walls [45], misaligned inlets for the fluid components to be mixed [46], and three-dimensional turns [47]. 

In this computational work, we report on the effect on mixing when non-rectangular cross-sections are used for the curved sections forming a simple serpentine channel. The use of this type of section as its orientation along the channel is changed allows for the centers of rotation of the primary Dean vortexes that form to be shifted between the mixing units. The effect is akin to that achieved in Stroock micromixers [31,48], where the use on the floor of the straight channels of asymmetric ridges with variable apex positions is exploited to achieve extensional flows and induce chaotic advection. From an implementation point of view, the designs proposed retain the benefits associated with serpentine micromixers such as simple fabrication, easy cleaning, and potentially damage-free processing of biological samples containing large molecules. The numerical work discussed is performed for a range of Reynolds numbers from *Re* = 1 to 100 for both the new design and the design of the standard serpentine micromixer.

## 2. Geometrical Design of the Micromixer 

**Figure 1:**
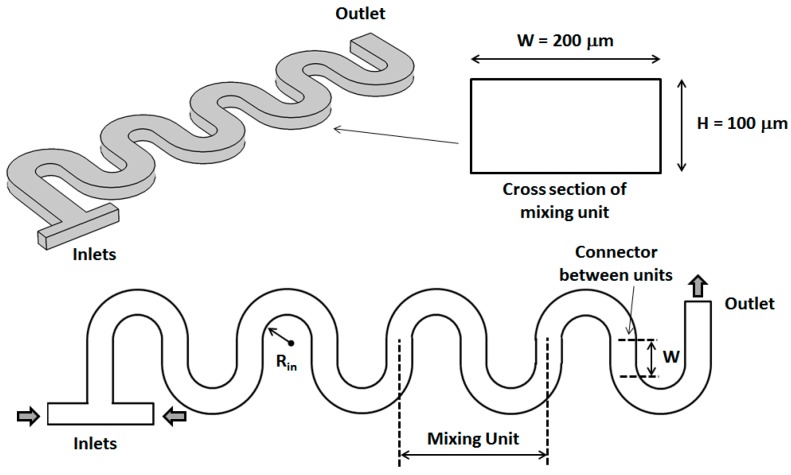
3D and top views of the standard serpentine micromixer with a rectangular cross-section defined by *W =* 200 μm and *H* = 100 μm.

**Figure 2:**
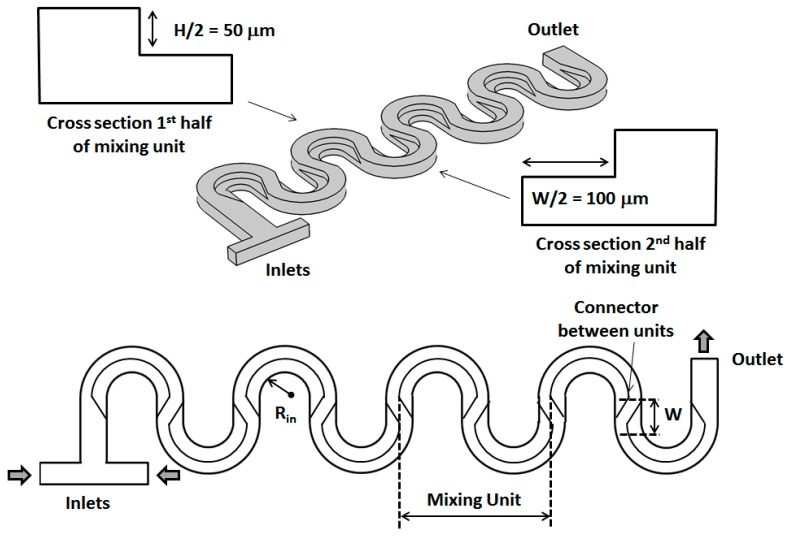
3D and top views of the new serpentine micromixer design employing non-rectangular cross-sections. As shown above, the orientation of the cross-section of the mixer is changed after each turn of the serpentine.

The basic designs of serpentine micromixers used in this computational study are shown in Figure 1 and Figure 2. The fluids to be mixed are fed into the mixer through a T-shaped inlet structure. The shape of both inlets is square with a size of 50 μm. For both designs, each mixing unit consists of two semicircular sections connected by a straight one. For all the channels investigated, the total height of the main channels is *H* = 100 μm, while its total width is *W* = 200 μm. The length of the straight sections connecting subsequent turns is set to be equal with *W*. For the non-rectangular cross-section designs, the height of the thinner part is maintained constant at *H*/2 = 50 μm, while its width is set at *W*/2 = 100 μm. As shown in Figure 2, between each turn of the serpentine, the orientation of the non-rectangular cross-section is changed so that the thicker part of the channels is always on the outside of the turns. For this study, the fluid flow and mixing performance have been analyzed for values of the inner turn radius *R_in_* corresponding to 0.5 *W* for both the standard rectangular section micromixer as well as for the newly investigated design.

## 3. Numerical Model and Mixing Assessment

The flow fields for each channel are obtained by solving the Navier–Stokes equations of motion for an incompressible Newtonian fluid in steady state pressure-driven flow:

(1) $$\rho \left[\frac{\partial u}{\partial t}+\left(u\cdot \nabla \right)u\right]=-\nabla p+\eta \nabla^{2}u$$

(2) ∇·u=0

where ***u*** (m∙s^−1^) is the velocity vector, *ρ* (kg∙m^3^) is the fluid density, *η* (kg∙m^−1^∙s^−1^) is the fluid viscosity, *t* (s) is the time, and *p* (Pa) is the pressure. The flow field equations are solved using a generalized minimal residual method (GMRES) iterative solver with a geometrical multigrid pre-conditioner and a Vanka algorithm for the pre- and post-smoothing. No-slip boundary conditions were set for the walls of the micromixer. A free tetrahedral mesh is used for the entire microchannel with no less than ~200,000 elements for all the geometries studied.

The fluid speeds and pressures thus obtained are then used to compute the species concentration throughout the micromixers using the convection-diffusion equation:

(3) $$\frac{\partial c}{\partial t}=D\nabla^{2}c-u\cdot \nabla c$$

where *c* (mol∙m^−3^) is the concentration of the species of interest, and *D* (m^2^∙s^−1^) is its diffusion constant, respectively. The same iterative numerical solver as for the Navier–Stokes equations is used, but the maximum element size in the mesh is constrained to less than 10 μm through the whole geometry to avoid the possible numerical errors that can be associated with this type of solution. Thus, for all the concentration simulations, the number of mesh elements in the numerical model has never been less than ~1,900,000. For all the simulations described in this work, we used the computational package COMSOL Multiphysics 5.1 (COMSOL Inc., Stockholm, Sweden) and its computational fluid dynamics/chemical engineering module. The accuracy of the numerical work employed for this type of flow has been previously validated against data obtained from other microfluidic devices [49,50]. In particular, when comparing model data with measured data on similar planar curved microchannels, as presented by Jiang et al. [50], we find mixing times to be less than 0.05 s for Dean numbers equal to or above 140, in agreement with the experimental results.

To quantify the mixing quality, we use a mixing index based on calculating the Shannon entropy associated with the distribution of the various components across sections perpendicular to the microchannels. Previous work has shown this type of measure to be consistent with other mixing measures and, at the same time, to be more mathematically rigorous and easily adaptable to the various formats in which computational and experimental data is presented, such as concentration distributions, particle tracer Poincaré maps, or fluorescent intensity images [51,52]. In all of these cases, the data of interest is converted to image data, and the informational complexity of these images is then analyzed to quantify the level of segregation or mixing of the components of interest.

In brief, to quantify the mixing, the obtained cross-sectional concentration distributions at different positions along the channel are first converted to 8-bit grayscale intensity maps [53]. These images are divided into a number *N_bins_* of equal size regions. For a system with two components, the mixing index is defined as

(4) $$M=-\frac{1}{ln2}\cdot \frac{1}{N_{bins}}\cdot \overset{N_{bins}}{\underset{j=1}{\sum }}\left[p_{1/j}ln\left(p_{1/j}\right)+p_{2/j}ln\left(p_{2/j}\right)\right]$$

where *p*_1/*j*_ and *p*_2/*j*_ are the conditional probabilities for Components 1 and 2, respectively, to be located in bin *j*. They represent the fraction of Components 1 and 2, respectively, in each bin relative to the total. They are calculated as the ratio of the average bin grayscale intensity from the corresponding concentration image, normalized by the maximum intensity, i.e., 255 for grayscale image data. In this particular study, since we have two chemical species and the fluids used are incompressible the two conditional probabilities are related as *p*_2/*j*_ *=* 1 − *p*_1/*j*_*.* As shown in Equation (4), the mixing index *M* is normalized by a factor of *ln*2, where 2 corresponds to the number of components. Thus, the mixing index will take the value *M* = 0 for completely segregated components, while it will assume the value *M* = 1 for the completely mixed case.

## 4. Results and Discussion

For the analysis of all the mixers, water solutions have been considered as the working fluids, each with a density *ρ* of 1000 kg∙m^−3^ and a viscosity *η* of 0.001 kg∙m^−1^∙s^−1^. The diffusion coefficient *D* was fixed to 1.0 × 10^−9^ m^2^∙s^−1^ corresponding to the diffusion values for most ions in aqueous solutions. For all the simulations, the working solutions were considered to be pure water introduced through one of the inlets and dyed water with a concentration *c* = 1 moL∙m^−3^ introduced through the opposite inlet. The flow rates are maintained for both fluid components, with the same mean fluid velocity at both inlets. This means fluid velocity is varied from 0.0075 to 0.75 m∙s^−1^ to span a range of Reynolds numbers corresponding to *Re* = 1–100. 

**Figure 3:**
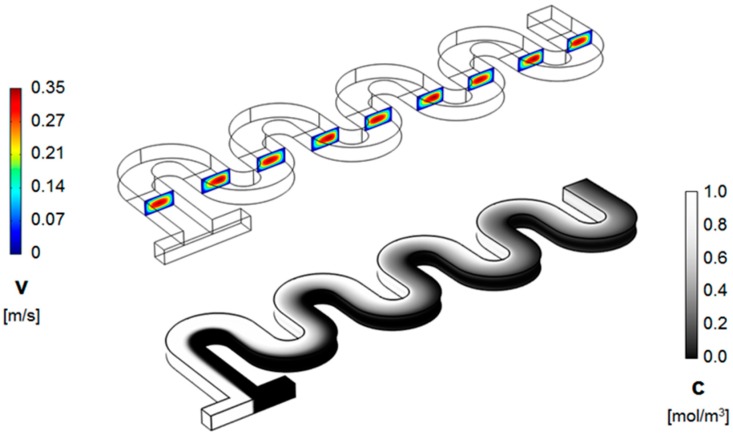
Velocity magnitude (cross-sectional maps) (**top**) and concentration distribution (surface map) (**bottom**) along the channel of a standard serpentine micromixer (*R_in_* = *W*/2 = 100 μm, and *Re* = 20).

**Figure 4:**
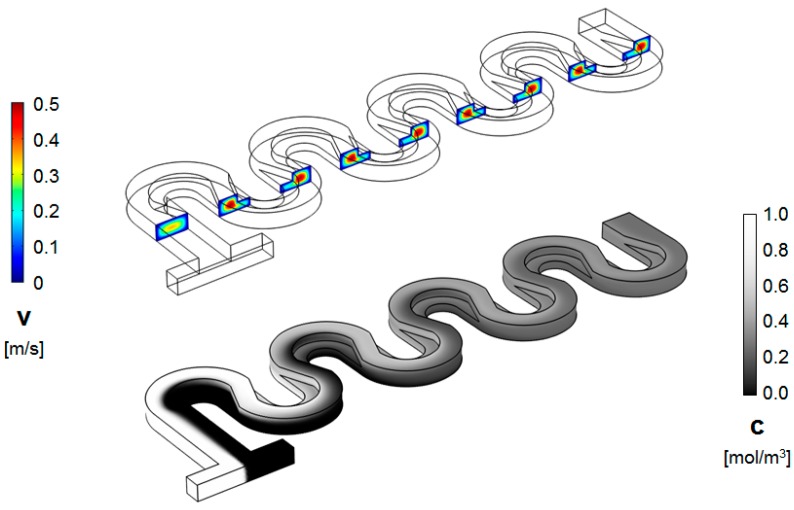
Velocity magnitude (cross-sectional maps) (**top**) and concentration distribution (surface map) (**bottom**) along the channel of a non-rectangular cross-section serpentine micromixer (*R_in_ = W/2* = 100 μm, and *Re* = 20).

Representative results for the flow fields and concentration distributions for the designs investigated are shown in Figure 3 and Figure 4. From the evolution of the concentration along the length of the micromixer, it is immediately apparent that its distribution is distinct between the two designs. 

**Figure 5:**
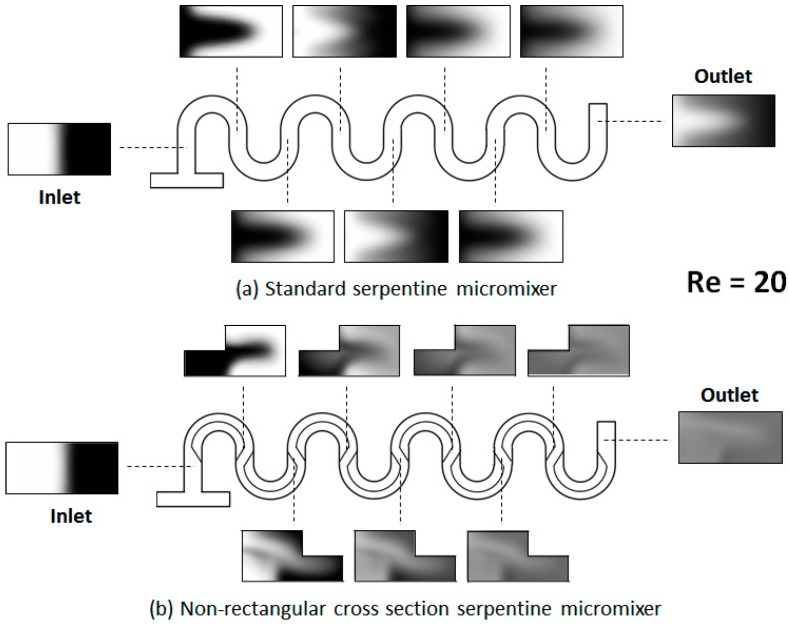
Transversal concentration distribution at various positions along the channel for (**a**) a standard serpentine micromixer and (**b**) a serpentine micromixer with a non-rectangular cross-section (*Re* = 20). The concentration is mapped at both the midpoint and the end of each mixing unit.

**Figure 6:**
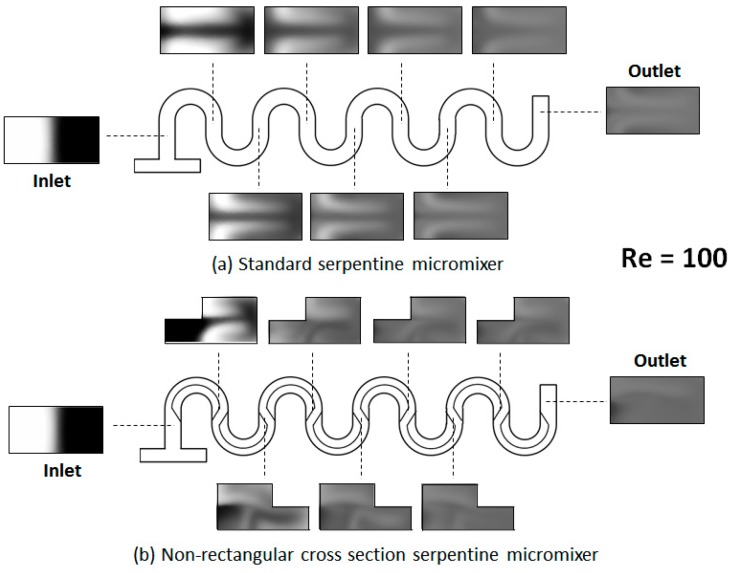
Transversal concentration distribution at various positions along the channel for (**a**) a standard serpentine micromixer and (**b**) a serpentine micromixer with a non-rectangular cross-section (*Re* = 100).

As shown in the figures above, in the standard design, the two fluids, which are introduced in the system through the opposite ends of the T-joint inlet, remain on distinctive sides of the serpentine through the length of the device, for Reynolds numbers as high as *Re* = 30. On the other hand, for the serpentine with non-rectangular cross-sections, that change orientation between the serpentine sections, the concentration distribution starts to homogenize after the first mixing unit. This conclusion from the concentration surface maps is supported by the cross-sectional concentration maps. As shown in Figure 5 and Figure 6, the concentration distribution transversal to the flow illustrate different behaviors in the two designs. While, in both microchannel types, fluid motion does occur transversally as expected under the presence of Dean flows in curved geometries [18], in the regular serpentine channel, the two fluids remain mostly separated. The interface between the fluid streams does deform, which increases the contact area between them, but the mixing remains limited to the molecular diffusion at the boundary. In contrast to the modified design, aside from the interface stretching, sizable segmentation of the fluid streams also occurs, at Reynolds numbers as low as *Re* = 20. In fact, based on the evolution of the concentration along the channel (Figure 5 and Figure 6), as soon as the third mixing cycle occurs, there are virtually no pockets of low (*c* = 0 mol∙m^−3^) and high (*c* = 1 moL∙m^−3^) concentration, respectively, left in the dyed fluid distribution map. 

**Figure 7:**
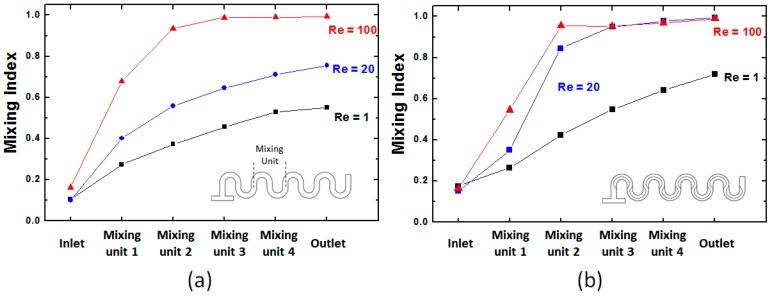
Position dependence of the mixing index along serpentine micromixers at different Reynolds numbers: (**a**) a standard serpentine microchannel and (**b**) a modified channel.

This visual assessment of the concentration distribution in the two types of micromixers is also confirmed by the quantitative analysis of the mixing achieved in them. Graphs of the mixing index (Figure 7) indicated that, as the fluids flow along the channel, their intermixing increases. Additionally, increasing the fluid rates (Reynolds number) is associated with better mixing performance. The mixing increase with Re is consistent with previous analyses of Dean flow micromixers [18,52], which showed that the Dean vortices that formed in curved microchannels become increasingly complex and undergo bifurcations at high Reynolds numbers, which are associated with the onset of chaotic advection. However, while, in the regular serpentine micromixer, full mixing (*M* = 1) is not achieved except in the high Reynolds number regime (*Re* = 100), the modified designs show very good mixing behavior across the full range of inlet fluid rates investigated. In the non-rectangular cross-section mixers, full mixing is realized within three mixing units down to Reynolds numbers *Re* = 20. This corresponds to a mixing time of less than 0.035 s for Reynolds numbers larger than 20. Moreover, even at Reynolds numbers as low as 1, no mixing saturation is observed, indicating that full mixing is achievable by adding more mixing units to the channel.

From the mixing quality dependence on the position along the channel, it also has to be noted that, while in the standard serpentine channel, the rate of increase in the mixing index is almost monotonic as a function of the length of the channel, this is not the case for the non-rectangular section designs. Essentially after the first mixing unit for Reynolds numbers larger than *Re* = 20, there is a steep increase in mixing quality. This behavior is similar to that encountered in grooved chaotic advection micromixers [31], where changing the center of the transversal rotation of the fluids between mixing units leads to extensional flows that increase both the inter-lamination of the components to be mixed and their cross-flow. Previous work on this effect in mixers systems with asymmetric slanted groove (also known as staggered herringbone micromixers) [33] has found similar steep power law type increases in mixing performance, indicative of the onset of chaotic advection. While the mechanism for generating pressure-driven transversal flows is different, i.e., using slanted grooves with respect to the flow direction, versus constraining the fluid to move along curved channels, the idea of the approach used remains the same.

**Figure 8:**
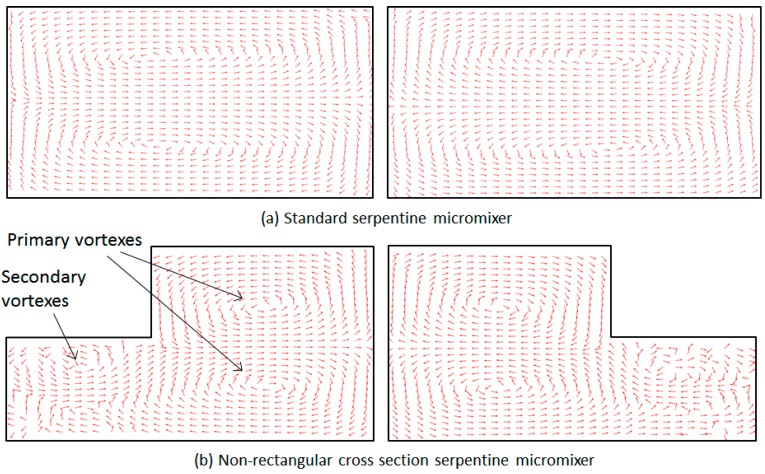
Arrow plots of the velocity field for the two mixers design: (**a**) a standard micromixer and (**b**) a non-rectangular cross-section micromixer. Both sets of data are collected for the 2nd mixing cycle at *Re* = 20. The left plots are for data collected at the midpoint of the first leg of the mixing cycle, while the right images are the corresponding plots for the second leg of the mixing cycle.

Arrow plots based on the velocity fields in the serpentine-based designs confirm that the confinement of trajectories along the curved lines induces counter rotating transversal flows. As shown in Figure 8, for the non-rectangular cross-section microchannels, the topology used allows for the center of rotation of these vortices to be changed between each half mixing unit. More importantly, the topology used leads to the generation of secondary vortexes once the second mixing cycle begins. While secondary vortexes can be created in regular curved Dean micromixers, their onset is associated with large Reynolds numbers, typically *Re* > 150 [31]. Our analysis indicates that the use of the proposed geometry allows the observed bifurcation of multiple vortexes to occur at much lower Reynolds numbers, providing a method of increasing the mixing performance of this type of device that is easy to implement. The proposed devices can be fabricated using soft-lithography techniques [13,54] based on replica molding transfer to polydimethylsiloxane (PDMS) from silicon stamps prepared using two-layer lithography. Due to the smaller non-rectangular cross-section of the channels, the pressure gradient in these devices will be a factor of ~1.9 larger than in their standard counterparts; nevertheless, this remains within the working range of PDMS-based devices.

## 5. Conclusions

In this work, we have investigated the use of a simple adaptation of serpentine-type micromixers in which channels with non-rectangular cross-sections are used. Numerical analysis of the quality of mixing that can be achieved in these micromixers indicates that the use of this topology, coupled with the change in orientation of the channel cross-section between mixing units, increased the mixing performance in terms of a low and intermediate Reynolds numbers for micromixers based on Dean flows. The simplicity of the modification proposed makes it easy to integrate typical serpentine designs used in microfluidic devices, whose applicability was limited by the large Reynolds numbers needed for efficient mixing. Future work will focus on optimization in terms of mixing efficiency of the geometrical parameters used (the size of the non-rectangular cross-section, the radius of curvature of the mixing units, and the length of the connector between them) as well as on the inlet conditions (the ratio of the flows and the nature of fluid components entering the micromixer).
